# LncRNA NONHSAT141924 promotes paclitaxel chemotherapy resistance through p-CREB/Bcl-2 apoptosis signaling pathway in breast cancer

**DOI:** 10.7150/jca.39463

**Published:** 2020-03-26

**Authors:** Ming Gu, Wenhui Zheng, Mingdi Zhang, Xiaoshen Dong, Yan Zhao, Shuo Wang, Haiyang Jiang, Xinyu Zheng

**Affiliations:** 1 Department of Breast Surgery, The First Affiliated Hospital of China Medical University, Shenyang, Liaoning 110001, People's Republic of China; 2 Department of anesthesiology, The Shengjing Hospital of China Medical University, Shenyang, Liaoning 110001, People's Republic of China; 3 Department of Breast Surgery, Obstetrics and Gynecology Hospital of Fudan University, Shanghai, 200011, People's Republic of China; 4 Lab 1, Cancer Institute, The First Affiliated Hospital of China Medical University, Shenyang, Liaoning 110001, People's Republic of China

**Keywords:** LncRNA, paclitaxel-resistance, p-CREB/Bcl-2 apoptosis signaling pathway, breast cancer

## Abstract

Breast cancer is the most prevalent malignant neoplasm among women worldwide. Despite continuous improvement of breast cancer individualized comprehensive therapy, local recurrence and distant metastasis still remain the challenges due to the development of acquired drug-resistance. Long non-coding RNAs (LncRNAs) is known to participated in the development of breast cancer. However, the mechanisms of LncRNAs involving in drug-resistance of breast cancer during chemotherapy remain to be further elucidated. Aiming to screen for candidate LncRNAs responsible for breast cancer mechanism, we first investigated the expression patterns of LncRNAs and mRNAs in paired breast cancer tissues and normal tissues using Agilent Human lncRNA array. The microarray results clearly demonstrated multiple differentially expressed mRNAs and LncRNAs including LncRNA NONHSAT141924. The remarkable up-regulation of LncRNA NONHSAT141924 in breast cancer MCF-7 was further confirmed by quantitative real-time PCR. GO and KEGG pathway analysis demonstrated that LncRNA NONHSAT141924 was most closely associated with paclitaxel (PTX)-resistant phenotype. To further explore the mechanism by which LncRNA NONHSAT141924 contributes to PTX-resistant characteristics, LncRNA NONHSAT141924 was transfected into MCF-7 breast cancer cell line. Overexpression of LncRNA NONHSAT141924 significantly reduced MCF-7 cell survivability through modulation of p-CREB/Bcl-2 apoptosis signaling pathway, one of the major pathways participated in LncRNAs-mediated chemotherapy resistance. Taken together, our study provides a new LncRNA-mediated regulatory mechanism for PTX-resistance of breast cancer and suggests that therapeutic inhibition of LncRNA NONHSAT141924 might be an efficient strategy for PTX-resistant breast cancer treatment.

## Introduction

Breast cancer is one of the leading lethal malignancies among women worldwide 1. Clinical responses to antitumor therapies are often limited to a subset of patients 2. Current strategies of breast cancer therapy include surgical resection and subsequent chemotherapy. The standard first-line chemotherapy has long been applied due to its superior efficacy and low toxicity. Despite its excellent and durable responses in the vast majority of cases, patients subjected to chemotherapy frequently experienced relapse within few years due to the development of acquired drug resistance. The current scenario strongly underlines the urgency to identify innovative and more effective molecular therapeutic targets for drug resistance of breast cancer. Although various studies have focused on drug resistance in breast cancer chemotherapy, the specific molecular mechanisms are still unclear. Thus, it is considerably interest to explore potential biomarkers that are significantly associated with drug resistance in breast cancer chemotherapy.

Long non-coding RNAs (lncRNAs) are a large class of non-protein-coding transcripts with length of > 200 bp. Although cumulative evidence has shown that lncRNAs are frequently deregulated in various diseases and are involved in numerous pathophysiological and biological processes such as proliferation, apoptosis, metastasis, and cell migration^3^, ^4^, only a small numbers of lncRNAs have been characterized functionally^5^, ^6^. For instance, Liu et al. identified that LncRNA NKILA represses NF-kB signaling and cancer-associated inflammation via binding to NF-kB/IkB complex 7 and low NKILA expression is associated with breast cancer metastasis and poor patient prognosis 8. However, the specific roles of lncRNAs in mediating drug-resistance and regulating breast cancer progression are not well studied.

In this study, we screened the expression profiles of lncRNAs and mRNAs in breast cancer samples and their corresponding adjacent normal tissue samples and identified multiple unique differentially expressed lncRNAs and mRNAs using microarray technology. Applying biological information analysis, we demonstrated that LncRNA NONHSAT141924 was remarkably upregulated in breast cancer tissues. GO and KEGG pathway analyses identified that LncRNA NONHSAT141924 potentially played a key role in paclitaxel (PTX)-resistance during breast cancer pathogenesis. Our findings further showed that therapeutic inhibition of LncRNA NONHSAT141924 restrained PTX-resistance through p-CREB/Bcl-2 apoptosis signaling pathway, the major pathway involved in chemotherapy resistance for breast cancer. In conclusion, our study might provide a novel mechanism and potential therapeutic target for PTX-resistance breast cancer.

## Materials and Methods

### Tissue specimens

A total of 35 primary breast infiltrating ductal carcinoma tissues and their paired normal tissues were surgically obtained at the First Affiliated Hospital of China Medical University. All cases were diagnosed both clinically and pathologically, and have not been preoperatively treated with chemotherapy, radiation therapy or endocrine therapy. After surgery, fresh tissues were snap-frozen in liquid nitrogen and stored at -80°C for total RNA extraction. Written informed consent was obtained from all study participants. This study was approved by the Ethics Committee of Human Study of China Medical University.

### Microarray analysis

Total RNAs were isolated from three breast cancer tissues of patients with lymphatic metastasis and three corresponding normal tissues and quantified by NanoDrop ND-2000 (Thermo Scientific). RNA integrity was assessed using Agilent Bioanalyzer 2100 (Agilent Technologies). According to the manufacturer's standard protocols, mRNA samples were reverse-transcribed into cRNA and labeled with cyanine-3-CTP. The cyanine-3-CTP-labeled cRNA samples were hybridized onto the lncRNA microarray chips (Agilent Human lncRNA, 4*180K). After washing, the arrays were scanned by Agilent Scanner G2505C (Agilent Technologies). The obtained array images were analyzed using Feature Extraction software (version10.7.1.1, Agilent Technologies) to extract raw data, which were further analyzed using GeneSpring. To begin with, the raw data were normalized with the quantile algorithm. The probes with flags in “P” in at least 1 out of 2 conditions were chosen for further data analysis. Genes or lncRNAs with fold change ≥ 2.0 and *p* value ≤ 0.05 were considered as the differentially expressed ones. To understand the function of lncRNAs associated with the breast cancer, the Gene Ontology (GO) and KEGG analyses were performed to study the potential functions of the dysregulated lncRNAs and mRNA. Finally, network interaction of lncRNAs in the breast patients was constructed. At last, hierarchical clustering was performed to display the distinguishable expression pattern of these genes between samples.

### Cell culture and transfection

Six human breast cancer cell lines (BT-549, MDA-MB-231, Hs-578T, ZR-75-30, T-47D and MCF-7) were obtained from the Cancer Institute of the First Affiliated Hospital, Shenyang, Liaoning Province, and cultured in DMEM medium (Gibco) supplemented with 10% fetal bovine serum (FBS, Gibco), 100 mg/ml streptomycin and 100 U/ml penicillin at 37°C in a humidified incubator in an atmosphere of 5% CO_2_.

MCF-7 cells were transfected with shRNA-LncRNA NONHSAT141924 or pcDNA-LncRNA NONHSAT141924 using Lipofectamine™ 2000 (Invitrogen, Carlsbad, CA, USA) according to the manufacturer's instructions. In brief, the transfection complexes were prepared by incubating LncRNA NONHSAT141924 shRNA/pcDNA and transfection reagent at room temperature for 25 min and added into the culture media. After 6 h of incubation, the media were replaced with fresh media and cells were further cultured till paclitaxel treatment or harvest.

### Paclitaxel treatment

Paclitaxel (Selleckchem) was prepared as a 10 μM stock solution in DMSO. Briefly, 1x10^6^ cells were plated in 100-mm culture dishes. After 24 hours of incubation, cells were treated with 20 nM paclitaxel for a total of 5 days, with media refreshing at 3 days of treatment. After rinsed with PBS, cells were maintained in drug-free culture with media replacement every 48 hours until proliferative cell clones were established.

### MTT assay

MTT assay was preformed using the Cell Growth Determination Kit (Sigma) as previously described 9. Briefly, cells were seeded onto 96-well plates at 1x10^3^ cells/well. After 24 hours of culture, cells were transfected with shRNA-LncRNA NONHSAT141924 or pcDNA-LncRNA NONHSAT141924 and treated with paclitaxel in 100 µL media. After 48 hours of treatment, 10 µL MTT (methylthiazol tetrazolium) solution was added into the media. After incubation at 37°C for 4 hours, the supernatants were removed, and cells were incubated with 100 µL of MTT solvent. Absorbance at 570 nm was spectrophotometrically measured using a BioTek ELx800. The assays were performed independently and repeated at least three times.

### Wound healing assay

Transfected cells were seeded onto 6-well plates. After cultured to 90% confluence, cells were wounded by drawing a scratch line with the same width using a sterilized tip on the bottom of each well. At 72 hours after wounding, cell images around the scratch line were captured. Each experiment was repeated in triplicates.

### Cell migration assays

24-well Transwell plates containing permeable polyethylene terephthalate membrane inserts with 8 µm pores (Invitrogen) were used. A total of 1x10^5^ transfected cells in 100 µL FBS-free DMEM were placed on the top layer of the Transwell inserts. The bottom chambers were filled with DMEM containing 10% FBS. After 48 hours of incubation, non-migratory cells were removed from the top surface of the membrane and the cells that passed through the membrane were fixed with methanol and stained with 0.5% crystal violet. The number of migratory cells was counted using an inverted light microscope (Nikon).

### RNA preparation, RT and qPCR

Total RNA from cells was extracted using Trizol reagent (Invitrogen). The RT and qRT-PCR reactions were performed as described previously 10, and the actin mRNA level was used as an internal control for normalization. The PCR primers used were as follows: 5'-AAAGACCTGTACGCCAACAC-3'(forward) and 5'-GTCATACTCCTGCTTGCTGAT-3' (reverse) for actin, 5'-GAGTCCTTTCTCCTCCAC-3' (forward) and 5'-TATTCCATACCTGCCAAC-3' (reverse) for NONHSAG014936, 5'-GAACCCTTGGCAGGCAGAA-3' (forward) and 5'-GACAGGCAGCAGGTATTGG-3' (reverse) for NONHSAT022406, 5'-GCGGCAGCATAGATGACA-3' (forward) and 5'-CCAACAAGGGAATGGAACA-3' (reverse) for NONHSAT141924, 5'-CAGTATCTCCCTCCTCACCTCA-3' (forward) and 5'-GGGCACCAGCCTCAAATC-3' (reverse) for NONHSAT089596, 5'-CTCTGGCAGGCTCTTTCTCC-3' (forward) and 5'-CTCCCTGTCAATGAAATCTGG-3' (reverse) for NONHSAG020595, and 5'-GAAGGCTCTGACACTCCACC-3' (forward) and 5'-ACGGGACACTTCTTCTCATG-3' (reverse) for FR278154. The relative mRNA expression changes were calculated using the 2^-ΔΔCt^ method.

### Western blot analysis

Transfected cells were harvested and lysed in lysis buffer (Beyotime Biotech, Shanghai, China) and the lysates were subjected to Western blot analysis as described previously 11.

### Statistical analysis

All statistical analyses were performed using SPSS version 19.0 software. All data are presented as mean ± standard deviation. The differences between two groups were analyzed using the Student t test. A p value less than 0.05 was considered statistically significant. All experiments were independently repeated at least three times.

## Results

### LncRNAs and mRNAs expression profiles in breast cancer

**Figure 1:**
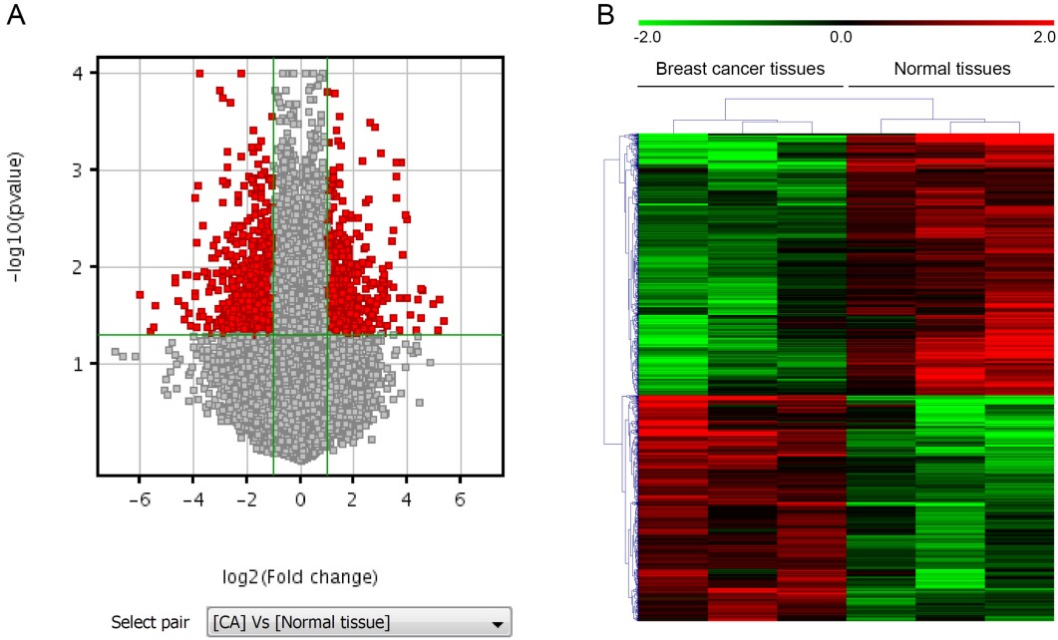
** LncRNAs and mRNAs expression profiles in breast cancer. A.** Volcano plot, where the two vertical lines are the 2-fold change boundaries and the horizontal line is the statistical significance boundary (p < 0.05). Genes with fold change ≥ 2 and statistical significance are marked with red dots. **B.** Heat map and hierarchical clustering of lncRNA profile comparison between the breast cancer and paired normal breast samples. Each row represents one lncRNA, and each column represents one tissue sample. The relative lncRNA expression is depicted according to the color scale. Red indicates up-regulation and green indicates down regulation. The numbers 2.0, 0 and -2.0 are folds changes in the corresponding spectrum.

To search for LncRNAs and mRNAs that are preferentially expressed in human breast cancer tissues and might contribute to breast cancer progression, we assessed the profiles of LncRNAs and mRNAs that are differentially expressed in breast cancer tissues and their paired normal tissues from three adult individuals with axillary lymph node metastasis using Agilent Human lncRNA array. The differentially expressed lncRNAs and mRNAs identified by microarray analysis. LncRNAs profiling detected 861 lncRNAs that were differentially expressed in breast cancer tissues with at least two-fold changes. Among them, 368 were up-regulated and 493 were down-regulated. Similarly, mRNAs profiling detected 544 mRNAs that were differentially expressed with at least two-fold changes in breast cancer tissues compared with their paired normal tissues. Among them, 282 were up-regulated and 262 were down-regulated. Differentially expressed lncRNAs and mRNAs were selected by volcano plot filtering (fold change ≥ 2 and P-value ≤ 0.05) as shown in Figure 1A. Hierarchical clustering of expression of the 861 lncRNAs and 544 mRNAs based on centered Pearson correlation clearly separated breast cancer from normal tissues (Figure 1B).

### Go and KEGG pathway analyses

To predict the functions and explore potential biological associations of the differentially expressed lncRNAs, the 861 differentially expressed lncRNAs and 544 mRNAs were subjected to GO (GO Biological Process, GO Cellular Component, GO Molecular Function) and KEGG pathway analyses. The enriched functional terms were considered as their predicted functional terms. The results of GO molecular function analysis indicated that these lncRNAs and mRNAs are enriched in several functional pathways. Among them, ATP binding, sequence-specific DNA binding transcription factor activity, and sequence-specific DNA binding were most closely associated with breast cancer (Figure 5A). GO biological process analysis revealed that these lncRNAs and mRNAs are involved in signal transduction (Figure 5B). GO cellular component analysis showed that these lncRNAs and mRNAs are mostly enriched in extracellular region (Figure 5C). Furthermore, KEGG pathway analysis showed that these lncRNAs and mRNAs are involved in some specific pathways, including pathways in cancer, cytokine-cytokine receptor interaction, systemic lupus erythematosus, etc. (Figure 5B). Among these lncRNAs and mRNAs, LncRNA NONHSAT141924 was most closely associated with PTX-resistant phenotype.

### Validation of the microarray data by qRT-PCR

**Figure 2:**
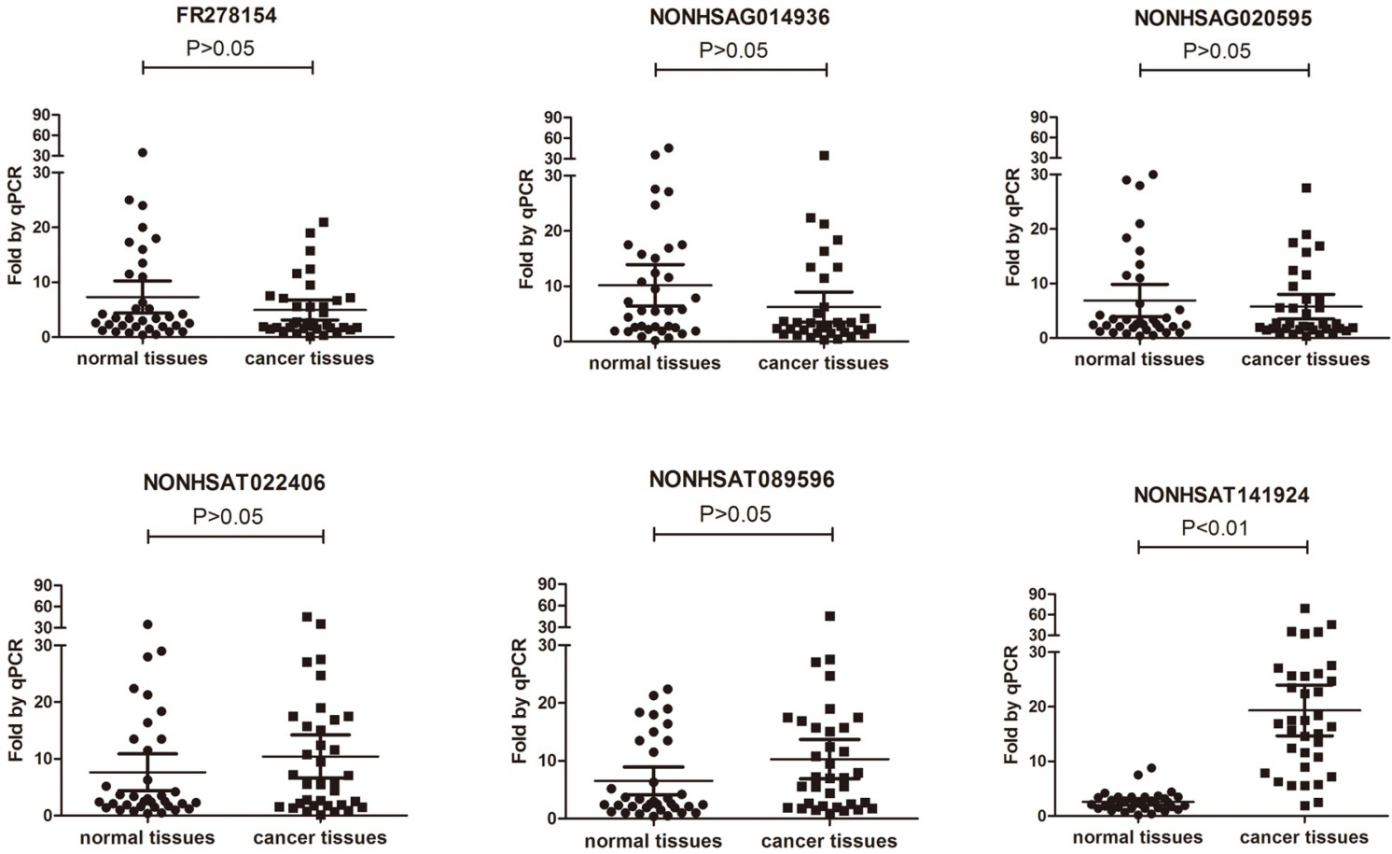
** Comparison between microarray data and qRT-PCR results.** Differential expression of NONHSAG014936, NONHSAT022406, NONHSAT089596, NONHSAG020595, FR278154 and NONHSAT141924 identified by microarray between 3 paired breast cancer samples and normal tissue samples was validated by qRT-PCR in 35 paired tissues. The expression levels of lncRNAs were normalized to that of U6.

To validate the results of microarray, 3 down-regulated lncRNAs and 3 up-regulated lncRNAs (Table 1) were randomly selected and their expression levels in the breast cancer tissues and their paired normal tissue of 35 patients with breast cancer were further compared using qRT-PCR (Table 2). The results showed that only the expression of LncRNA NONHSAT141924 was significantly higher in the breast cancer samples than in the paired normal tissue samples (Figure 2).

**Figure 3:**
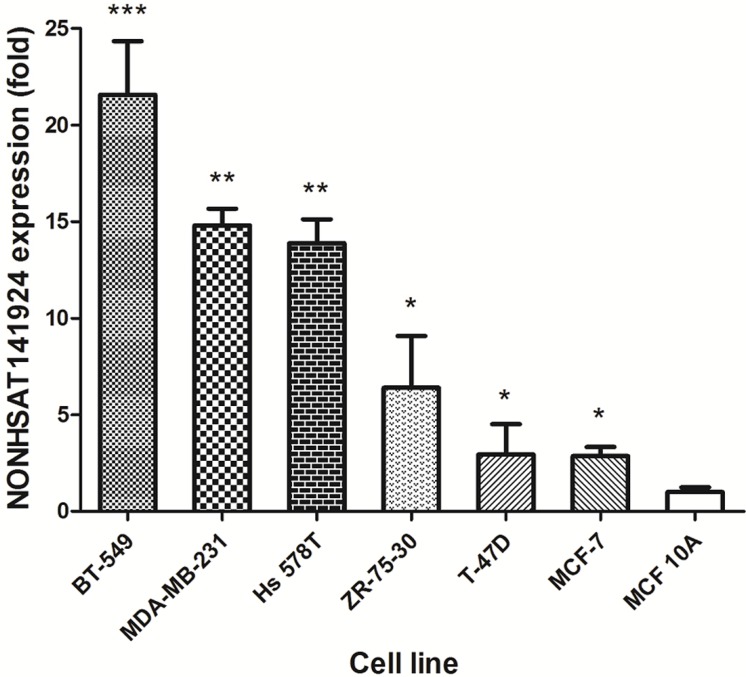
Validation of NONHSAT141924 expression in breast cancer cell lines.

Furthermore, we explored the expression of LncRNA NONHSAT141924 in breast cancer cell lines and normal breast epithelium cell line. Consistently, we found that LncRNA NONHSAT141924 expression was remarkably higher in six breast cancer cell lines (BT549, MDA-MB-231, Hs578T, ZR-73-30, T47D, MCF-7) than in normal breast epithelium cell lines (MCF-10A) (Figure 3). Considering that the expression of LncRNA NONHSAT141924 was significantly enhanced in both breast cancer cell lines and breast cancer tissues, we then focused on LncRNA NONHSAT141924 later.

### LncRNA NONHSAT141924 promotes the proliferation but not migration of breast cancer *in vitro*

**Figure 4:**
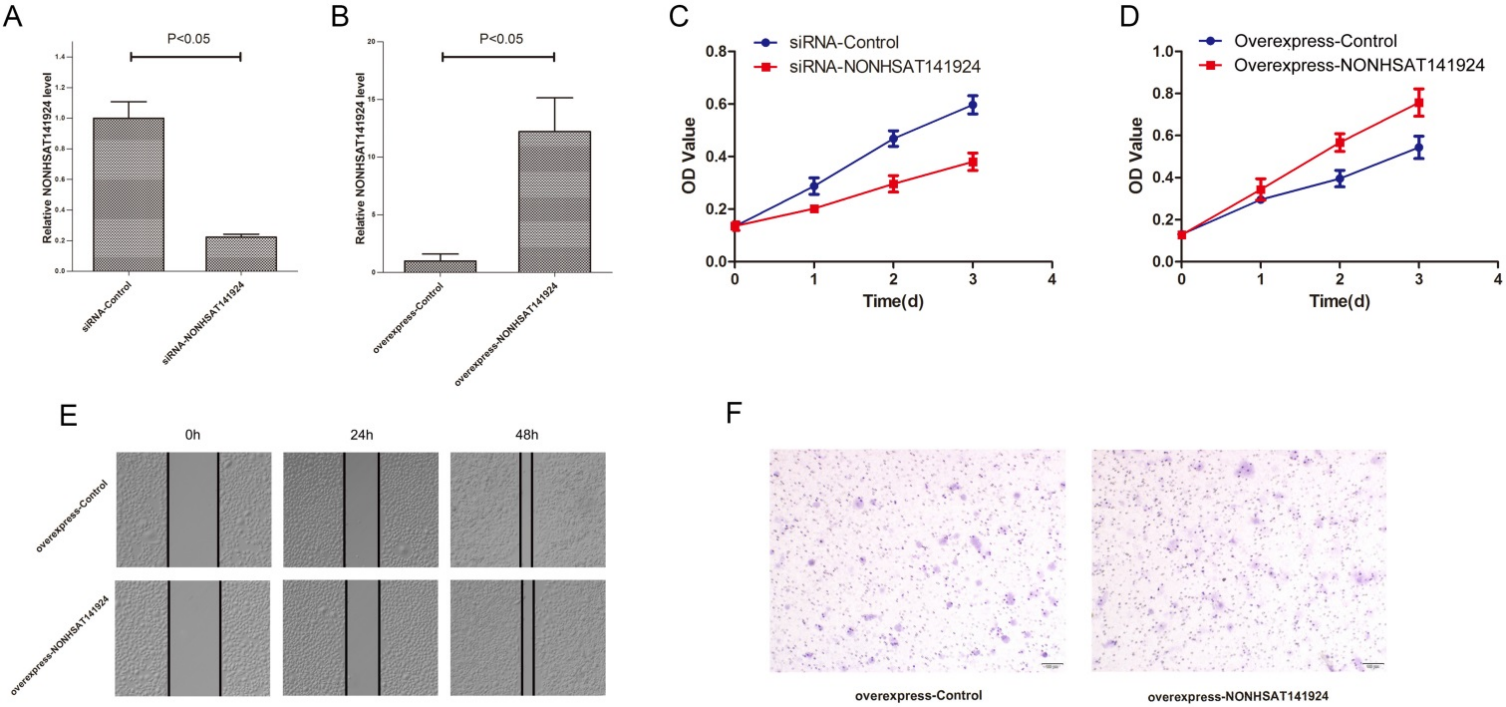
** LncRNA NONHSAT141924 promotes proliferation but not migration and invasion of breast cancer *in vitro*.** Efficiencies of LncRNA NONHSAT141924 inhibition (**A**) and overexpression (**B**) in MCF-7 cells. **C.** The results of MTT assays showing the cell growth rates of LncRNA NONHSAT141924-overexpressed or -downregulated MCF-7 cells. The results of wound healing assay (**E**) and Transwell migration assay (**F**) showing the migration of MCF-7 cells with LncRNA NONHSAT141924-overexpression and down-regulation.

**Figure 5:**
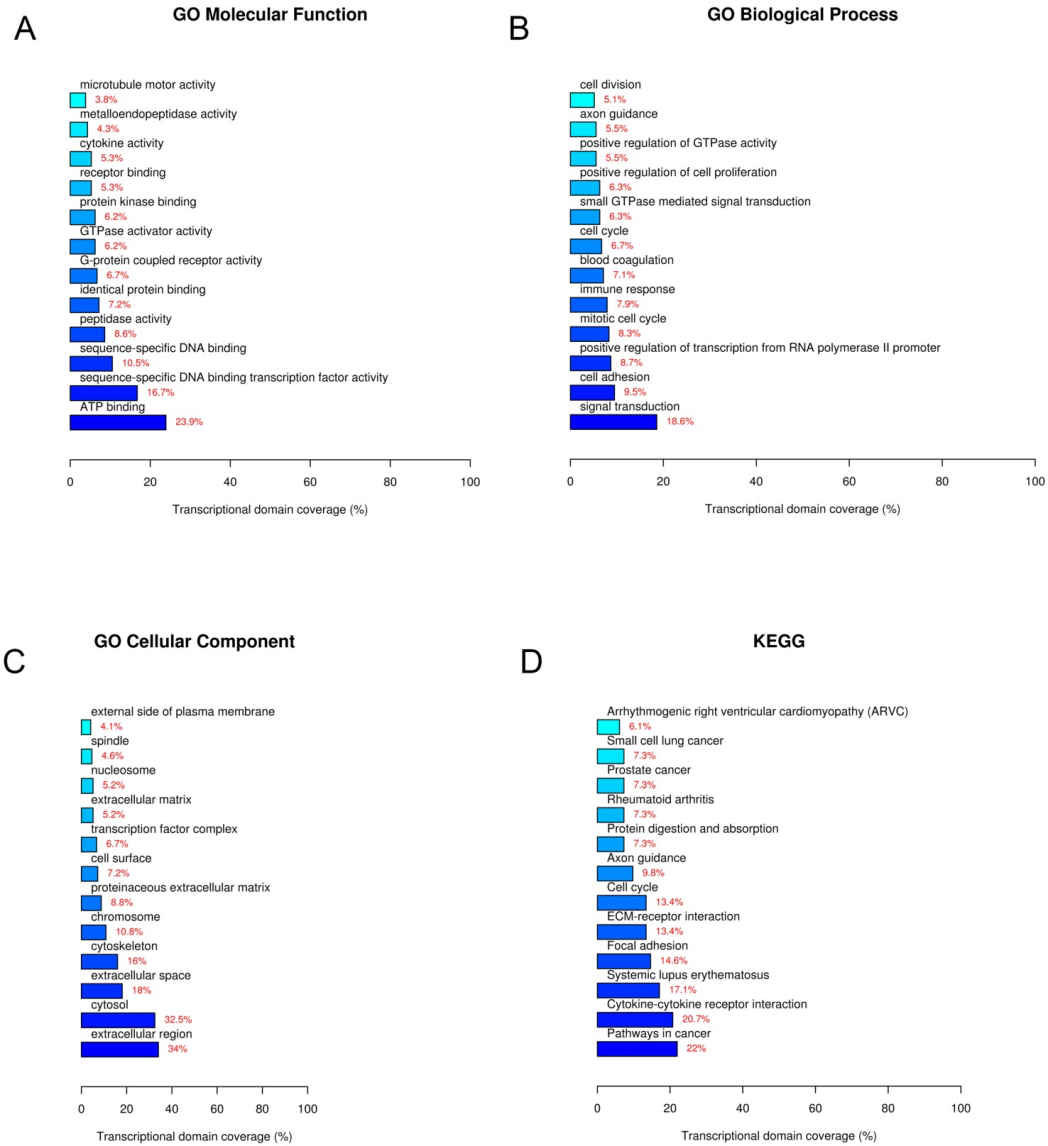
** GO and KEGG pathway analyses. A.** GO molecular function. **B.** GO biological process. **C.** GO cellular component. **D.** KEGG pathway.

LncRNA NONHSAT141924 is 536 nucleotides long and located in human chromosome 16, which is evolutionarily conserved among several species. To explore the effects of LncRNA NONHSAT141924 on breast cancer proliferation and migration, we overexpressed and knocked down LncRNA NONHSAT141924 in breast cancer cell line MCF-7 (Figure 4A and B). MTT assay indicated that LncRNA NONHSAT141924 significantly promoted breast cancer cell proliferation (Figure 4C and D). By contrast, wound healing and Transwell assays demonstrated that LncRNA NONHSAT141924 had no obvious effect on the migration of breast cancer cells compared with normal breast cells (Figure 4E and F). Overall, our results indicate that LncRNA NONHSAT141924 can significantly promote breast cancer cell proliferation but not migration *in vitro*.

### LncRNA NONHSAT141924 promotes PTX-resistance through p-CREB/Bcl-2 apoptosis signaling pathway in breast cancer cells

**Figure 6:**
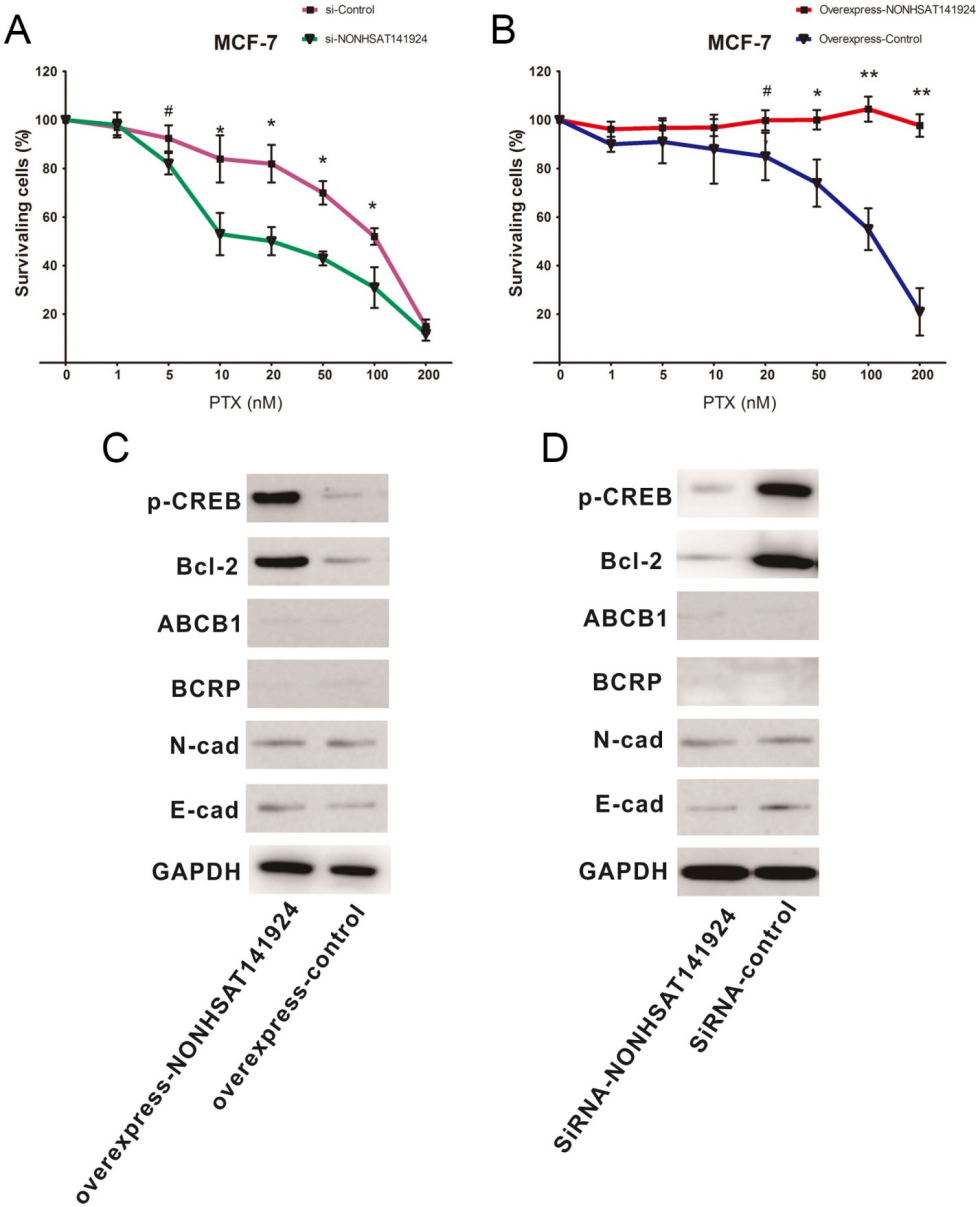
** LncRNA NONHSAT141924 promotes PTX-resistance through p-CREB/Bcl-2 apoptosis signaling pathway in breast cancer. A.** The survival rates of cells with LncRNANONHSAT141924 overexpression or knockdown and the blank control cells as measured using MTT assay under treatment of PTX with concentration ≥ 20nM (P < 0.05). **B.** The images of Western blot analysis showing the levels of Bcl-2, p-CREB, ABCB1, BCRP, E-cadherin, N-cadherin proteins in NONHSAT141924-overexpressed and knocked down cells.

To further identify the role of LncRNA NONHSAT141924 in PTX-resistance characteristics of breast cancer, LncRNA NONHSAT141924 was overexpressed or knocked down in MCF-7 breast cancer cells. MTT assay clearly showed that under PTX exposure, LncRNA NONHSAT141924 overexpression significantly increased cell survival rate while LncRNA NONHSAT141924 down-regulation decreased cell survival rate (Figure 6A and B).

It has been reported that LncRNAs are involved in chemotherapy resistance mainly through three mechanisms. First, they participate in apoptosis of tumor cells through pathways mediated by Bcl-2 and its upstream transcription factor p-CREB. Second, they regulate drug metabolic pathways by ABCB1- and BCRP-mediated reduction of drug accumulation in tumor cells. Third, they are involved in epithelial-mesenchymal transition by modulating E-cadherin and N-cadherin mainly through Wnt/β-Catenin and AKT/mTOR signaling pathways 12-14. To further investigate which pathway is involved in LncRNA NONHSAT141924 induced PTX-resistance, LncRNA NONHSAT141924 was overexpressed or knocked down in MCF-7 cell line (Figure 4A and B). Western-blot analysis demonstrated that overexpression of LncRNA NONHSAT141924 increased the levels of Bcl-2 and p-CREB proteins while knockdown of LncRNA NONHSAT141924 decreased the levels of Bcl-2 and p-CREB proteins. By comparison, both overexpressed or knockdown of LncRNA NONHSAT141924 had no significant effects on the levels of ABCB1, BCRP, E-cadherin and N-cadherin proteins (Figure 6C and D).

## Discussion

The present study demonstrated that LncRNAs were differentially expressed both *in vivo* and *in vitro* in breast cancer and could play essential roles in chemotherapy resistance. Specifically, we identified LncRNA NONHSAT141924 as a novel LncRNA that positively regulates PTX-resistance. Furthermore, our results suggest that therapeutic inhibition of LncRNA NONHSAT141924 in breast cancer cells may significantly reduce cell survival rate and even reverse PTX-resistance phenotype by exerting an anabolic effect.

Breast cancer is one of the leading lethal malignancies among women worldwide. However, the pathogenesis of breast cancer still remains unclear 15. Current strategies of breast cancer therapy include surgical resection and subsequent chemotherapy. The standard first-line chemotherapy has long been applied due to its superior efficacy and lower toxicity 16. Despite the excellent and durable responses in the vast majority of cases, patients subjected to chemotherapy frequently experienced relapse within few years due to the development of acquired drug resistance. The current scenario strongly underlines the urgency to identify novel and more effective molecular therapeutic targets for drug resistance in breast cancer 17. Although various studies have focused on drug resistance in breast cancer chemotherapy, the specific molecular mechanism is still unclear. Therefore, further exploring potential biomarkers which are significantly associated with drug resistance in breast cancer chemotherapy is of great importance.

Emerging evidence has indicated that multiple LncRNAs are important regulators of cancer-related genes 18-21. However, few LncRNAs have yet been identified to contribute to drug resistance in cancer. In our study, we found that remarkably increased LncRNA NONHSAT141924 level is accompanied with breast cancer progression both *in vitro* and *in vivo*. This observation provides a clinical insight into the contribution of LncRNANONHSAT141924 to the pathophysiological regulation of breast cancer progression during chemotherapy resistance. In the present study, LncRNA NONHSAT141924 was markedly upregulated in breast cancer tissues compared with normal tissues as shown by Agilent Human lncRNA array. This result was further validated in six breast cancer cell lines and 35 breast cancer patients. MTT assay indicated that LncRNA NONHSAT141924 could significantly promote proliferation of breast cancer cells, whereas wound healing and Transwell assays demonstrated it had no obvious effect on the migration of breast cancer cells.

Bioinformatics analysis further clarified the inconsistency that LncRNA NONHSAT141924 can significantly promote breast cancer cell proliferation but not migration *in vitro*. GO and KEGG pathway analysis identified that LncRNA NONHSAT141924 was most closely associated with PTX-resistance phenotype. Paclitaxel is a first-line drug in tumor chemotherapy nowadays and drug resistance of tumor cells to paclitaxel is a common cause of chemotherapy failure 22, 23. Our study demonstrated that LncRNA NONHSAT141924 significantly increased cell survival rate under PTX exposure. To further investigate the pathway involved in LncRNA NONHSAT141924 induced PTX-resistant, we overexpressed/knocked down LncRNA NONHSAT141924 to mimic the high/low LncRNA NONHSAT141924 conditions in the MCF-7 BC cell line. Western-blot analysis demonstrated that overexpression of LncRNA NONHSAT141924 increased Bcl-2 and p-CREB protein levels and knockdown of LncRNA NONHSAT141924 decreased Bcl-2 and p-CREB protein levels, whereas both had no significant effect on the levels of ABCB1, BCRP, E-cadherin and N-cadherin proteins.

In summary, our study provides new findings that LncRNA NONHSAT141924 promotes paclitaxel chemotherapy resistance in breast cancer both *in vitro* and *in vivo*. LncRNA NONHSAT141924 functions through inhibiting p-CREB/Bcl-2 apoptosis pathway, one of the major pathways of chemotherapy resistance at present. These findings provide new insights into the possibility of using LncRNAs therapy for drug resistance in breast cancer. We anticipate that our study can provide a foundation for future investigations on the role of LncRNAs in regulating the breast cells' responses to chemotherapy and on the potentials of LncRNAs serving as an effective model system for studying gene regulation in the development of gene therapy for treating human cancers related to drug resistance.

## Figures and Tables

**Table 1:** The LncRNA of differential expression.

GeneSymbol	Breast cancer	Normal tissues	Fold change	p value
NONHSAG014936	1.900	4.598	0.154	0.036
NONHSAG020595	7.455	8.478	0.492	0.046
FR278154	4.151	6.225	0.238	0.017
NONHSAT022400	5.878	4.798	2.115	0.030
NONHSAT089596	8.342	6.839	2.834	0.021
NONHSAT141924	4.403	3.389	2.018	0.023

**Table 2:** Characteristics of patients (n=35).

Characteristic	N	No.(%)
**Age(yr)**		
<35	7	20
>35	28	80
**Gender**		
female	35	100
male	0	0
**Histology**		
Ductal	29	82.9
Lobular	6	17.1
**T stage**		
T1	8	22.9
T2	26	74.3
T3	1	2.8
**N stage**		
N0	0	0
N1	29	82.9
N2	6	17.1
**Histological grade**		
Ⅰ	0	0
Ⅱ	35	100
Ⅲ	0	0
**ER**		
-	20	57.1
+	15	42.9
**PR**		
-	17	48.6
+	18	51.4
**HER2**		
-	14	40
+	21	60
